# Randomized, multicenter, open-label trial of autologous cytokine-induced killer cell immunotherapy plus chemotherapy for squamous non-small-cell lung cancer: NCT01631357

**DOI:** 10.1038/s41392-020-00337-x

**Published:** 2020-10-19

**Authors:** Liang Liu, Quanli Gao, Jingting Jiang, Junping Zhang, Xin Song, Jiuwei Cui, Yunbin Ye, Zhiyu Wang, Xinwei Zhang, Xiubao Ren

**Affiliations:** 1 Department of Immunology and Biotherapy, Key Laboratory of Cancer Immunology and Biotherapy, Key Laboratory of Cancer Prevention and Therapy, Tianjin’s Clinical Research Center for Cancer, Tianjin Medical University Cancer Institute and Hospital, Tianjin, 300060 China; 2 Department of Immunotherapy, Affiliated Cancer Hospital of Zhengzhou University & Henan Cancer Hospital, Zhengzhou, 450008 Henan China; 3 Department of Tumor Biological Treatment, Third Affiliated Hospital of Soochow University, Changzhou, 213003 Jiangsu China; 4 Department of Oncology, Shanxi Bethune Hospital, Taiyuan, 030032 Shanxi China; 5 Cancer Biotherapy Center, Third Affiliated Hospital of Kunming Medical University (Tumor Hospital of Yunnan Province), Kunming, 650118 Yunnan China; 6 Cancer Center, First Hospital of Jilin University, Changchun, 130021 Jilin China; 7 Laboratory of Immuno-Oncology, Fujian Cancer Hospital & Fujian Medical University Cancer Hospital, Fuzhou, 350014 Fujian China; 8 Department of Immuno-Oncology, Fourth Hospital of Hebei Medical University, Shijiazhuang, 050011 Hebei China

**Keywords:** Health care, Immunotherapy

**Dear Editor,**

Cytokine-induced killer (CIK) cells have been recognized as a new type of anti-tumor effector cells. CIK cells are a mixture of T lymphocytes. Among them, CD3+ /CD56+ T cells, which are rare in uncultured peripheral blood, are the main effector cells. CIK cells can proliferate rapidly in vitro, with stronger antitumor activity, broader target tumor spectrum, and lower adverse effect than other reported antitumor effector cells.^1^ Their ease of production in vitro and antitumor potential have made them suitable candidates for cell therapy regimens in solid and hematopoietic tumor treatments.^1,2^ Our previous retrospective study showed that the median progression-free survival (PFS) and overall survival (OS) in untreated, advanced non-small-cell lung cancer (NSCLC) patients who received CIK cell immunotherapy plus chemotherapy (13 and 24 months, respectively) were significantly longer than in those who received chemotherapy alone (6 and 10 months, respectively).^2^ But so far, there is no prospective, multicenter clinical study in lung cancer. Based on our previous study, we designed this randomized, multicenter, open-label trial to further evaluate the clinical efficacy of CIK cell immunotherapy plus chemotherapy in patients with advanced squamous NSCLC (ClinicalTrials.gov number, NCT01631357).

Patients and methods were showed in Supplementary Appendix (Supplementary Materials and Methods). Between December 1, 2014, and December 31, 2018; a total of 111 patients from 8 university-affiliated hospitals in China were screened for randomization, there were 100 participants who met eligibility criteria (Fig. 1a). A total of 90 patients were assigned randomly to either the CIK cell immunotherapy plus chemotherapy group (CIK-CT group, *n* = 45) or the chemotherapy group (CT group, *n* = 45). In the CIK-CT group, thirty-nine patients completed four cycles of treatment. Six patients did not completed four cycles of treatment, including 4 patients due to the disease progression and 2 due to the intolerable adverse events. In the CT group, twenty-two patients completed four cycles of treatment. Twenty-three patients did not completed four cycles of treatment, including 16 patients due to the disease progression and 7 due to the intolerable adverse events. There was no patient lost to follow-up in the two groups. The baseline demographic and disease characteristics were generally well balanced between the two groups (Supplementary Table S1). Distributions of patients’ post-progression treatment in the two groups were showed in Supplementary Table S2. Follow-up was started from December 1, 2014, and ended on June 1, 2019.

**Fig. 1:**
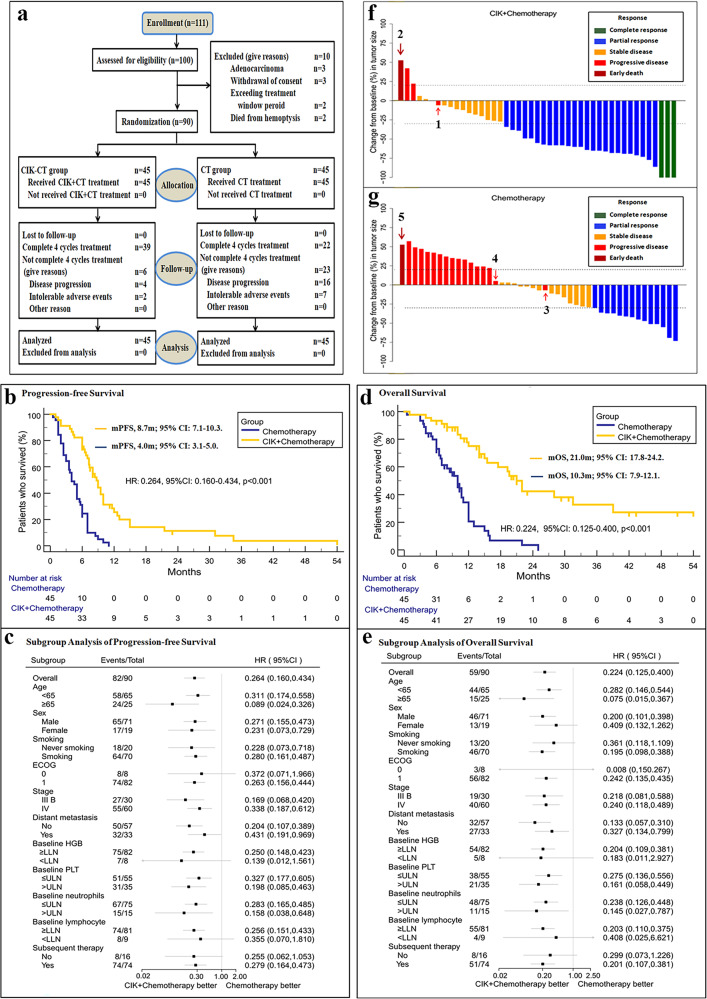
**a** NCT01631357 CONSORT Flow Diagram. CIK, CIK cell immunotherapy; CT, chemotherapy. **b** Kaplan–Meier estimates of progression-free survival in the two trial groups assessed by investigator according to Version 1.1 of The Response Evaluation Criteria in Solid Tumors. **c** Analysis of progression-free survival in prespecified subgroups. **d** Kaplan–Meier estimates of overall survival in the two trial groups assessed by investigator according to Version 1.1 of The Response Evaluation Criteria in Solid Tumors. **e** Analysis of overall survival in prespecified subgroups. **f** Change from baseline in sum of the longest diameters of target lesions in patients in CIK cell immunotherapy plus chemotherapy group, assessed by investigator according to Version 1.1 of The Response Evaluation Criteria in Solid Tumors (*N* = 45); 1, appearance of new malignant pleural effusion confirmed by pathology at firth effect evaluation; 2, 1 patient died from intestinal obstruction after 1 cycle treatment and not received therapeutic evaluation. **g** Change from baseline in sum of the longest diameters of target lesions in patients in chemotherapy group, assessed by investigator according to Version 1.1 of The Response Evaluation Criteria in Solid Tumors (*N* = 45); 3, appearance of new bone metastasis at first effect evaluation; 4, appearance of new liver metastasis at first effect evaluation; 5, 1 patient died from massive hemoptysis after 1 cycle treatment and not received therapeutic evaluation

In patients of CIK-CT group, the median count of untreated PBMCs was 18.0 × 10^8^ (range, 11.0 × 10^8^ to 31.5 × 10^8^) per cycle. The median count of autologous CIK cells after 15 days of amplification was 198.0 × 10^8^ (range, 105.0 × 10^8^ to 365.0 × 10^8^) per cycle. Results of phenotypic analysis of PBMCs and CIKs were showed in Supplementary Fig. S1.

The median PFS was 8.7 months (95% CI, 7.1 to 10.3) in the CIK-CT group and 4.0 months (95% CI, 3.1 to 5.0) in the CT group (HR, 0.26; 95% CI, 0.16 to 0.43; *P* < 0.001) (Fig. 1b). Subgroup analyses showed that the PFS in CIK-CT group was better than that in CT group in most of the sub-groups analyzed (Fig. 1c). The median OS was 21.0 months (95% CI, 17.8 to 24.2) in the CIK-CT group and 10.3 months (95% CI, 7.9 to 12.1) in the CT group (HR, 0.22; 95% CI, 0.13 to 0.40; *P* < 0.001) (Fig. 1d). Subgroup analyses showed that the OS in CIK-CT group was better than that in chemotherapy group in most of the sub-groups analyzed (Fig. 1e).

The ORR (CR + PR) was 62.2% (95% CI, 47.9 to 76.5%) in the CIK-CT group and 31.1% (95% CI, 17.4 to 44.8%) in the CT group (*P* < 0.001) (Supplementary Table S3). The change from baseline in the sum of the longest diameters of target lesions is shown in Fig. 1f, g. The median duration of response was 9.6 months (range, 1.5+ to 53.0+) in the CIK-CT group and 5.0 months (range, 1.5 to 10.0) in the CT group (Supplementary Fig. S2). Duration of exposure and first confirmed response in the two trial groups patients were showed in Supplementary Fig. S3.

Adverse events (AEs) of any grade were no difference between in the CT group (100%) and in the CIK-CT group (93.3%) (*P* = 0.356) (Supplementary Table S4). AEs of grade 3 or 4 occurred in 42.2% of the patients in the CT group and in 33.3% of the patients in the CIK-CT group (*P* = 0.296); led to the discontinuation of all treatment components in 15.6 and 4.4%, respectively (*P* = 0.157); and led to dose reduction of chemotherapy in 11.1 and 15.6% (*P* = 0.535). Distributions of adverse events induced by CIK cells or chemotherapy in CIK-CT group were showed in Supplementary Table S5.

In a review of the literature, we found that, to date, the current trial has been the first multicenter, randomized study of CIK cell immunotherapy plus chemotherapy in squamous NSCLC. The results of this phase II trial involving patients with untreated advanced, squamous NSCLC showed that the addition of CIK cell immunotherapy to standard chemotherapy with cisplatin and gemcitabine, as compared with chemotherapy alone, prolonged median OS by 10.7 months (21.0 months vs. 10.3 months) and median PFS by 4.7 months (8.7 months vs. 4.0 months). The risk of death was 78% lower and the risk of disease progression was 76% lower in the CIK cell immunotherapy plus chemotherapy group than in the chemotherapy group. Subgroup analyses showed that the OS and PFS in CIK cell immunotherapy plus chemotherapy group were better than those in chemotherapy group in most of the sub-groups analyzed. A higher response rate and longer duration of response were also observed in CIK cell immunotherapy plus chemotherapy group than in chemotherapy group.

Conventional chemotherapy can mediate tumor cell sensitivity to adoptive T-cell transfer. Chemotherapy can induce autophagy in tumor cells. Autophagosomes improve the mannose-6-phosphate receptor (MPR) accumulation on the membrane of tumor cells. MPR is able to bind granzyme B, which is released by activated CTLs, on the membrane of tumor cells.^3^ On the other hand, CIK cells can reverse chemoresistance. Our previous research showed that CIK cells could reverse the cisplatin (DDP) resistance of A549/DDP cell line in a time-dependent manner by reducing glutathione-S-transferase-π expression to increase the accumulation of DDP.^4^ Recently, Yost and colleagues showed that, through paired single-cell RNA and T cell receptor sequencing on 79,046 cells from site-matched tumors from patients with basal or squamous cell carcinoma before and after anti-PD-1 therapy, pre-existing tumor-specific T cells may have limited reinvigoration capacity, and that the T cell response to checkpoint inhibitor derives from a distinct repertoire of T cell clones that may have recently entered the tumor.^5^ CIK cells as exotic T cells may have stronger ability to kill tumor cells when compared with pre-existing tumor-infiltrating lymphocytes. These findings suggest that the addition CIK cell immunotherapy to chemotherapy can improve the efficacy of chemotherapy.

In summary, this phase II study provides evidence for the efficacy and safety of CIK cell immunotherapy plus chemotherapy in patients with previously untreated, advanced squamous NSCLC. A large sample, multi-center randomized, phase III trial is being carried out in our hospital to further validate these findings.

## Supplementary information

Supplementary Appendix
