# Supplementary material for: Randomized, multicenter, open-label trial of autologous cytokine-induced killer cell immunotherapy plus chemotherapy for squamous non-small-cell lung cancer: NCT01631357
Source: Signal Transduct Target Ther. 2020 Oct 19;5:244. doi: 10.1038/s41392-020-00337-x (PMC7572396; doi:10.1038/s41392-020-00337-x)
Supplement: Supplementary file 1 — Supplementary Appendix [file 41392_2020_337_MOESM1_ESM.doc]

Supplementary Materials for

Randomized, Multicenter, Open-label Trial of Autologous Cytokine-Induced Killer Cell Immunotherapy plus Chemotherapy for Squamous Non-Small-Cell Lung Cancer: NCT01631357

Liang Liu ^1^, Quanli Gao ^2^, Jingting Jiang ^3^, Junping Zhang ^4^, Xin Song ^5†^, Jiuwei Cui ^6^, Yunbin Ye ^7^, Zhiyu Wang ^8^, Xinwei Zhang ^1^, Xiubao Ren ^1^

*^1^ Department of Immunology and Biotherapy, Key Laboratory of Cancer Immunology and Biotherapy, Key Laboratory of Cancer Prevention and Therapy, Tianjin’s Clinical Research Center for Cancer, Tianjin Medical University Cancer Institute and Hospital, Tianjin 300060, China*

*^2^ Department of Immunotherapy, Affiliated Cancer Hospital of Zhengzhou University & Henan Cancer Hospital, Zhengzhou 450008, Henan, China*

*^3^ Department of Tumor Biological Treatment, Third Affiliated Hospital of Soochow University, Changzhou 213003, Jiangsu, China*

*^4^ Department of Oncology, Shanxi Bethune Hospital, Taiyuan 030032, Shanxi, China*

*^5^ Cancer Biotherapy Center, Third Affiliated Hospital of Kunming Medical University (Tumor Hospital of Yunnan Province), Kunming 650118, Yunnan, China*

*^6^ Cancer Center, First Hospital of Jilin University, Changchun 130021, Jilin, China*

*^7^ Laboratory of Immuno-Oncology, Fujian Cancer Hospital & Fujian Medical University Cancer Hospital, Fuzhou 350014, Fujian, China*

*^8^ Department of Immuno-Oncology, Fourth Hospital of Hebei Medical University, Shijiazhuang 050011, Hebei, China*

*†Deceased.*

*Correspondence to: Xiubao Ren (*[*renxiubao@tjmuch.com*](mailto:renxiubao@tjmuch.com)*) or Xinwei Zhang (zhangxinwei@tjmuch.com)*

This PDF file includes:

Materials and Methods

Supplementary Text

Figures S1 to S5

Tables S1 to S6

**Materials and Methods**

**Patients**

This study was approved by the State Food and Drug Administration of China (2006L01023), by the National Key Technologies R&D Program of China (2015BAI12B12 and 2018YFC1313400), and by the Ethical Committee of Cancer Hospital of Tianjin Medical University, according to the guidelines of the Declaration of Helsinki. Informed consent was obtained from all subjects. Patients who were eligible for enrollment had to meet the following criteria: age between 18 and 75 years, pathologically confirmed stage IIIB or IV squamous NSCLC (according to the seventh edition of the Cancer Staging Manual of the American Joint Committee on Cancer), not suitable for concomitant chemoradiotherapy, receiving no previous systemic therapy for advanced disease, an Eastern Cooperative Oncology Group (ECOG) performance status score of 0 or 1, at least one measurable lesion according to version 1.1 of the Response Evaluation Criteria in Solid Tumors (RECIST), and expected survival duration of ≥ 3 months. Patients were excluded if they had symptomatic central nervous system metastases, had immune deficiency or autoimmune diseases, had other malignancies, had severe allergic disorder, had uncontrollable medical condition, or were pregnant or lactating. We planned to complete all patients' recruitment within two years. However, the actual recruitment time was extended by two years due to the decrease in the incidence of advanced squamous NSCLC in China [Cheng, Y. *et al*. Real-world EGFR testing in patients with stage IIIB/IV non-small-cell lung cancer in North China: A multicenter, non-interventional study. *Thorac Cancer* **9**:1461-1469 (2018).]

**Trial design and treatment**

This phase 2 clinical study was a multicenter, randomized, open-label trial. The study was conducted at 8 university affiliated hospitals in China (Supplementary Table S6). Patients were assigned randomly, at a 1:1 ratio, to receive CIK cell immunotherapy plus chemotherapy (CIK-CT group) or chemotherapy (CT group). Random assignment was performed through a central telephone system using computer-generated, and permuted blocks with a block size of 4 and stratified according to study center.

As shown in Supplementary Fig. S4 and S5, all patients in the two groups received gemcitabine, 1000 mg/m^2^, on day 1 and 8, and cisplatin, 25 mg/m^2^, on day 1, 2 and 3; 4 weeks per cycle, for 4 cycles. In the CIK-CT group, patients also received autologous CIK cells infusion on day 15 and 16 (total count of CIK cells ≥ 1×10^10^), 4 weeks per cycle, for 4 cycles. After 4 cycles, all patients stop treatment to receive follow-up. Crossover between treatment arms was not allowed.

**End points and assessments**

The primary end point was PFS. PFS was measured from the date of randomization to the first disease progression or to death from any cause, whichever occurred first. The secondary end points included OS, objective response rate (ORR), the duration of response (DOR), and safety. OS was measured from the date of randomization until death from any cause. ORR was calculated by the percentage of patients with a confirmed complete (CR) or partial response (PR). DOR was measured from first documented complete or partial response to disease progression or death.

Patients were assessed by 2 independent experienced radiologists at each site using dynamic computed tomography or magnetic resonance imaging every 8 weeks from baseline for 12 months, and then every 3 months in both groups. If patients had clinical symptoms or aggravation symptoms during follow-up, they would receive imaging examination immediately. Response was determined based on the RECIST, version 1.1. Adverse events and abnormal laboratory findings were graded according to the National Cancer Institute Common Terminology Criteria for Adverse Events, version 3.0.

**CIK cells preparation**

CIK cells were prepared as described in our previous studies.[Liu, L. *et al*. Randomized study of autologous cytokine-induced killer cell immunotherapy in metastatic renal carcinoma. *Clin Cancer Res* **18,** 1751-1759 (2012). Li, R. *et al*. Autologous cytokine-induced killer cell immunotherapy in lung cancer: a phase II clinical study. *Cancer Immunol Immunother* **61,** 2125-2133 (2012)]. Brief description of CIK cells preparation was showed in Supplementary Fig. S5.

**Statistical analysis**

Sample size of the study was determined on the primary end point of PFS. According to the results of our previous, retrospective clinical study of autologous CIK cell immunotherapy plus chemotherapy in lung cancer [Li, R. *et al*. Autologous cytokine-induced killer cell immunotherapy in lung cancer: a phase II clinical study. *Cancer Immunol Immunother* **61,** 2125-2133 (2012)], the expected median PFS in the CT group was about 4.0 months and that in the CIK-CT group was about 9.0 months. The recruitment of patients would be completed in two years. Patients would be followed up for another year until the last participant was enrolled. The duration of the study would be three years. Assuming a 2-sided type I error of .05, a power of 80%, and considering the 10% drop-off rate, 96 patients were required given that we expect a hazard ratio (HR) of 0.44, which was estimated a 5-month increase (from 4 months to 9 months) in the median PFS. Ninety-six patients were randomly assigned, at a 1:1 ratio, into CT group or CIK-CT group.

Chi-square test and Fisher exact test were used for binary variable comparisons. Mann-Whitney U test was used for median comparisons. Distributions of survival time and duration of response were determined by Kaplan-Meier method. Associations between survival and potential prognostic factors were assessed using the log-rank test in univariable analyses. Median survival time along with Hazard ratios (HR) and associated 95% confidence intervals (CIs) were reported. Cox proportional hazards model was undertaken in multivariable analyses by using the Forward-LR (likelihood ratio) method with a significance level of .15 for entering and removing variables. In univariate evaluations of the prognostic impact of continuous variable, the optimal cutpoint was determined using the ROC Curve method, p-values were not adjusted for the optimization. All calculations were performed using R software version 3.5.1.

**
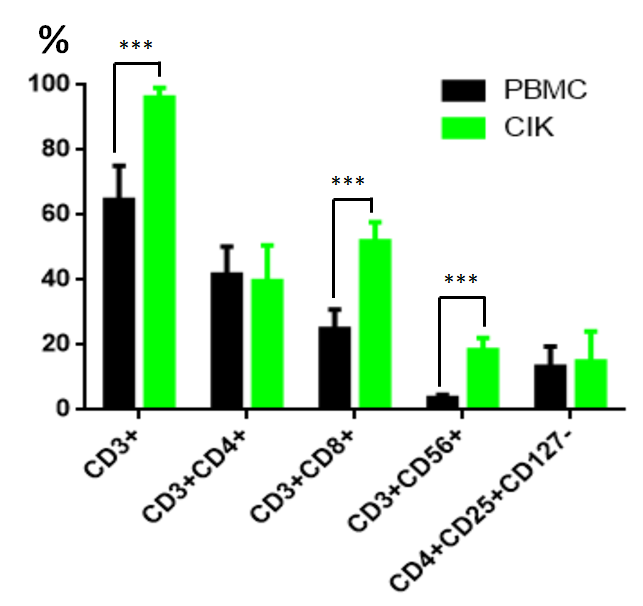
**

**Figure S1. Distribution of T Cell Subtypes in Peripheral Blood Mononuclear Cells (PBMCs) and CIK Cells in Patients Treatment with CIK Cell Immunotherapy plus Chemotherapy. ***, *P* < .001.**

Phenotypic analysis of PBMCs and CIKs were showed that the percentage of CD3+, CD3+CD4+, CD3+CD8+, CD3+CD56+ (NKT cells), CD4+CD25+CD127- (Treg cells) in PBMCs were 64.59% (95% CI, 54.00% to 75.18%), 41.74% (95% CI, 33.16% to 50.32%), 24.89% (95% CI, 18.84% to 30.94%), 3.59% (95% CI, 2.58% to 4.60%), and 13.28% (95% CI, 7.03% to 19.53%), respectively; and in CIKs were 96.24% (95% CI, 93.25% to 99.23%), 39.61% (95% CI, 28.60% to 50.62%), 51.95% (95% CI, 46.12% to 57.78%), 18.49% (95% CI, 14.84% to 22.14%), and 14.87% (95% CI, 5.59% to 24.15%), respectively. The percentages of CD3+, CD3+CD8+, and CD3+CD56+ cells in CIKs were significantly higher than those in PBMCs, while the percentage of CD3+CD4+, and CD4+CD25+CD127- cells had not significantly changed before and after culture.

**
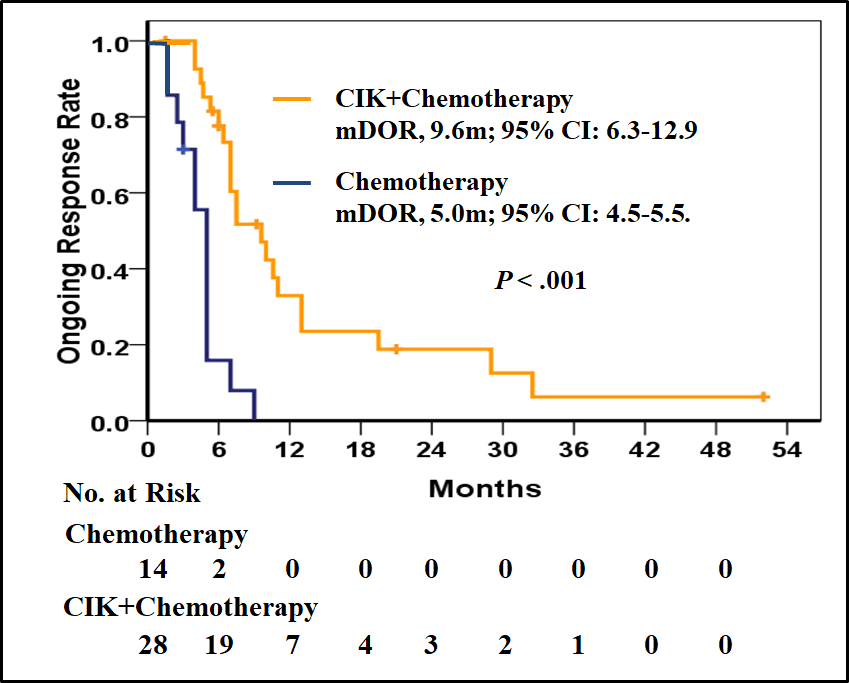
**

**Figure S2. Kaplan-Meier Estimates of Duration of Response in Patients of The Two Trial Groups. DOR, duration of response.**

**
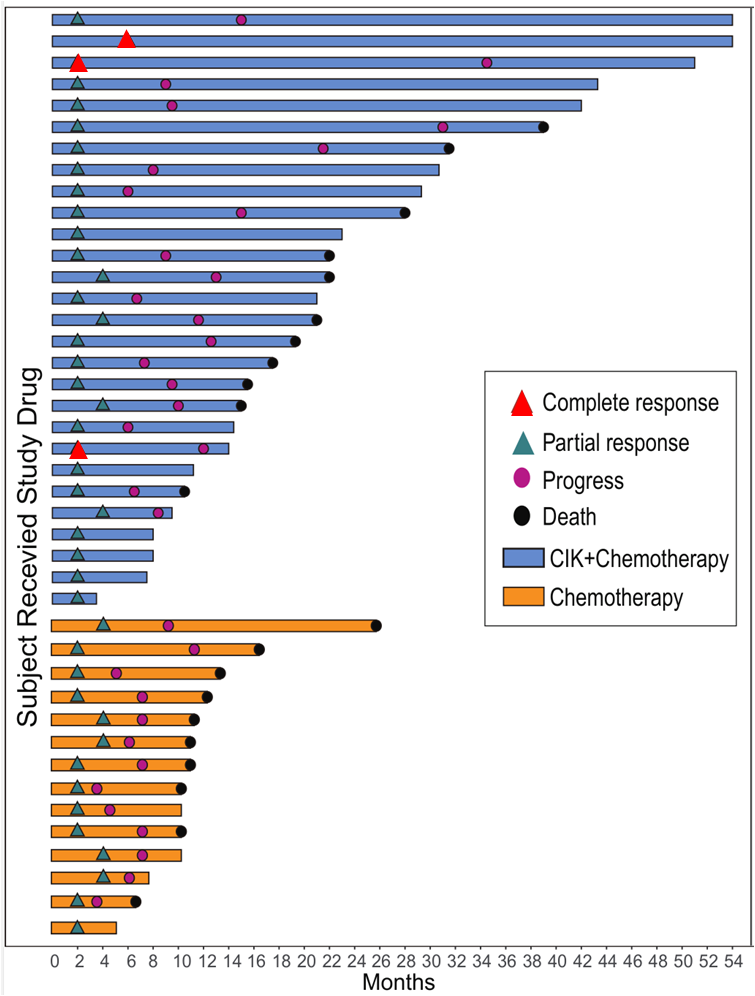
**

**Figure S3. Duration of Exposure and First Confirmed Response in The Two Trial Groups.**

**
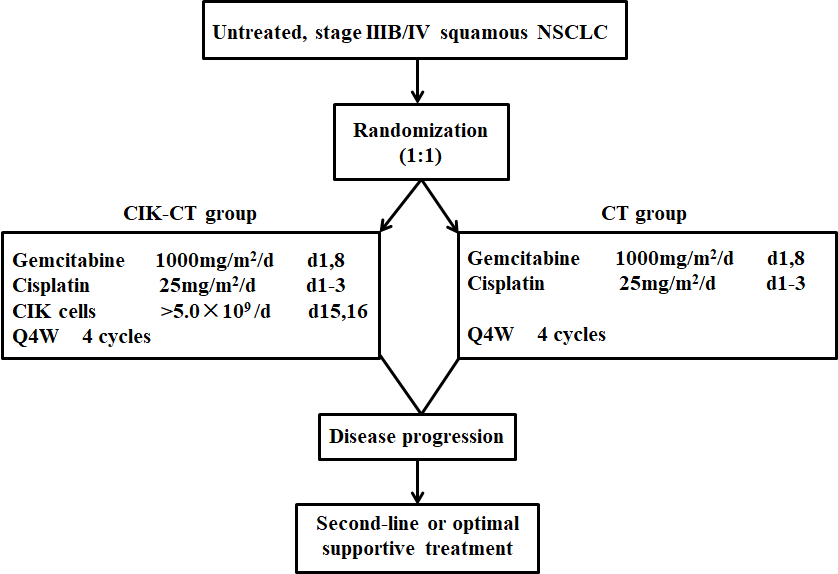
**

**Figure S4. Trial and Treatment Summary. NSCLC, non-small-cell lung cancer; CIK-CT, CIK cell immunotherapy plus chemotherapy; CT, chemotherapy.**

**
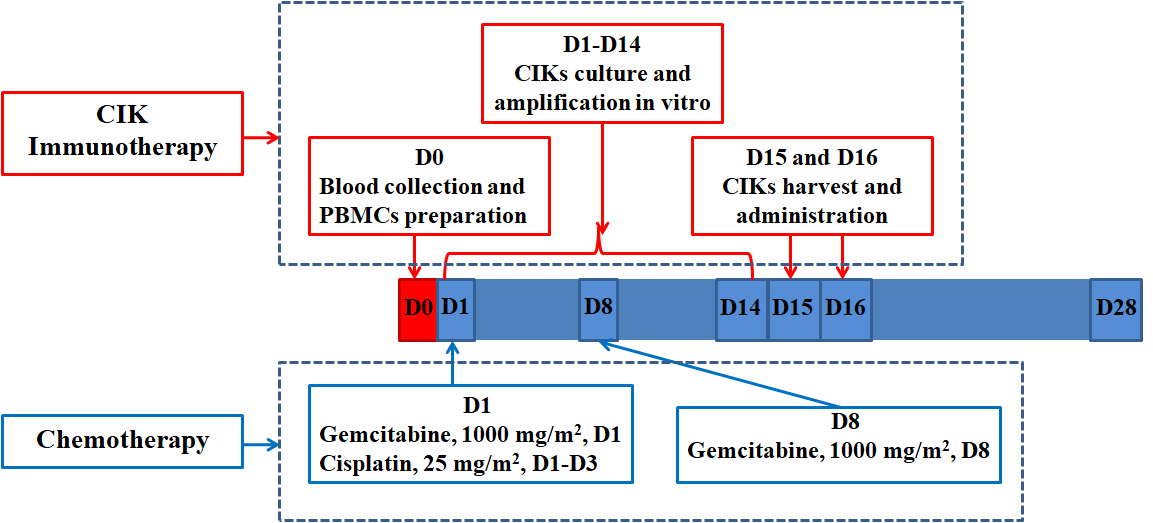
**

**Figure S5. Patient Treatment Schema Algorithm.**

CIK cells were prepared as described in our previous studies [Clin Cancer Res 18:1751-1759, 2012; Cancer Immunol Immunother 61:2125-2133, 2012]. Briefly, Peripheral blood mononuclear cell (PBMCs) were collected from patients using a Cobe Spectra Apheresis System (CaridianBCT, Lakewood, CO, USA), and cultured in X-VIVO 20 serum-free medium (Cambrex, East Rutherford, NJ, USA) containing 100 ng/mL anti-CD3 antibody (Ab) to stimulate CIK cells growth and 1000 IU/mL recombinant human interferon (IFN)-γ (Peprotech, Rocky Hill, NJ, USA), at 37 ºC with 5% CO_2_ for 24 h. Then, 1000 IU/mL recombinant human IL-2 (Peprotech) was added to the media. IL-2- and IFN-γ-containing medium was added to the culture system every 5 days. On day 15 and 16, CIK cells were harvested and analyzed for phenotype and cytotoxicity. Safety testing was performed during the course of cell culture. All products were free of bacterial and fungal contamination, negative for mycoplasma and contained < 5 Eu endotoxin. The phenotype and cytotoxicity of CIK cells were detected as described in our previous studies.

**Table S1.**

Demographic and Disease Characteristics of Patients at Baseline.

| **Characteristic** | **CIK-CT group**  **(N = 45)** | **CT group**  **(N = 4 5)** |
| --- | --- | --- |
| Male sex — No. (%) | 35 (77.8) | 36 (80.0) |
| Age |  |  |
| Median (Range) — years | 61 (36-73) | 61 (45-72) |
| <65 years — No. (%) | 32 (71.1) | 33 (73.3) |
| ECOG status score — No. (%) |  |  |
| 0 | 3 (6.7) | 5 (11.1) |
| 1 | 42 (93.3) | 40 (88.9) |
| Smoking status — No. (%) |  |  |
| Never | 11 (24.4) | 9 (20.0) |
| Former or current | 34 (75.6) | 36 (80.0) |
| Clinical stage — No. (%) |  |  |
| IIIB | 15 (33.3) | 15 (33.3) |
| IV | 30 (66.7) | 30 (66.7) |
| Distance metastasis — No. (%) |  |  |
| No | 29 (64.4) | 28 (62.2) |
| Yes | 16 (35.6) | 17 (37.8) |
| Hemoglobin — No. (%) |  |  |
| ≥LLN | 39 (86.7) | 43 (95.6) |
| <LLN | 6 (13.3) | 2 (4.4) |
| Platelets — No. (%) |  |  |
| >ULN | 18 (40.0) | 17 (37.8) |
| ≤ULN | 27 (60.0) | 28 (62.2) |
| Neutrophils — No. (%) |  |  |
| >ULN | 8 (17.8) | 7 (15.6) |
| ≤ULN | 37 (82.2) | 38 (84.4) |
| Lymphocytes — No. (%) |  |  |
| ≥ULN | 39 (86.7) | 42 (93.3) |
| <ULN | 6 (13.3) | 3 (6.7) |

Abbreviations: CIK-CT, CIK cell immunotherapy plus chemotherapy; CT, chemotherapy; ECOG, Eastern Co-operative Oncology Group; LLN, lower limit of normal; ULN, upper limit of normal.

**Table S2.**

**Distributions of Patients’ Post-progression Treatment in The** Two Groups.

|  | **CIK-CT Group** | **CT Group** |
| --- | --- | --- |
| **Patient State — No.** | **N=45** | **N=45** |
| No progression | 7^a^ | 1^b^ |
| Progression | 38^c^ | 44^d^ |
| **Post-progression treatment — No. ^e^** | **N=38** | **N=44** |
| No treatment | n=4 | n=4 |
| Yes treatment | n=34 | n=40 |
| Chemotherapy | 10 | 17 |
| Immunotherapy | 4 | 8 |
| Target therapy | 2 | 2 |
| Chemotherapy+immunotherapy | 1 | 2 |
| Chemotherapy+Target therapy | 6 | 2 |
| Chemotherapy+radiotherapy | 10 | 8 |
| Radiotherapy+Target therapy | 1 | 1 |

Notes: a, the 7 patients had completed four cycles of treatment; b, the 1 patient had completed four cycles of treatment; c, these patients included not completing four cycles of treatment because of intolerable adverse effects or disease progression, and completing four cycles of treatment; d, these patients included not completing four cycles of treatment because of intolerable adverse effects or disease progression, and completing four cycles of treatment; e, All patients not completing four cycles of treatment because of intolerable adverse effects in the two groups received subsequent therapy before disease progression.

**Table S3.**

**Summary of Response in Patients of The Two Trial Groups.**

| **Variable** | **CIK-CT group (N=45)** | **CT group (N=45)** |
| --- | --- | --- |
| Overall response — No. (%) | | |
| Complete response | 3 (6.7) | 0 |
| Partial response | 25 (55.5) | 14 (31.1) |
| Stable disease | 13 (28.9) | 14 (31.1) |
| Progressive disease | 3 (6.7) | 16 (35.6) |
| Not evaluable* | 1 (2.2) | 1 (2.2) |
| Objective response — No. (%) | 28 (62.2) | 14 (31.1) |
| Control of diseases — No. (%) | 41 (91.1) | 28 (62.2) |
| Duration of response — months | | |
| Median | 9.6 | 5.0 |
| Range | 1.5+ to 53.0+ | 1.5 to 10.0 |

Abbreviations: CIK-CT, CIK cell immunotherapy plus chemotherapy; CT, chemotherapy; *, In CIK-CT group, 1 patient died from intestinal obstruction after 1 cycle treatment and not received therapeutic evaluation, in CT group, 1 patient died from massive hemoptysis after 1 cycle treatment and not received therapeutic evaluation.

**Table S4.**

Distributions of Adverse Events in The Two Trial Groups.

| **Adverse Event** | **CIK+CT group (N=45)** | | | **CT group (N=45)** | |
| --- | --- | --- | --- | --- | --- |
|  | **Any Grade** | **Grade 3-4** | | **Any Grade** | **Grade 3-4** |
|  | **No.(%)** | | | | |
| Any adverse event | 42 (93.3) | 15 (33.3) | | 45 (100) | 19 (42.2) |
| Event leading to discontinuation of all treatment components | 2 (4.4) | 2 (4.4) | | 7 (15.6) | 7 (15.6) |
| Event leading to drug reduction | 7 (15.6) | 7 (15.6) | | 5 (11.1) | 5 (11.1) |
| Chemotherapy reduction | 7 (15.6) | 7 (15.6) | | 5 (11.1) | 5 (11.1) |
| CIK cell reduction | 0 | 0 | | 0 | 0 |
| Event leading to death | 0 | 0 | | 0 | 0 |
| Event | | | | | |
| Anemia | 29 (64.4) | 1 (2.2) | 37 (82.2) | | 0 |
| Nausea | 26 (57.8) | 3 (6.7) | 41 (91.1) | | 9 (20.0) |
| Vomit | 23 (51.1) | 3 (6.7) | 29 (64.4) | | 5 (11.1) |
| Thrombocytopenia | 18 (40.0) | 7 (15.6) | 20 (44.4) | | 10 (22.2) |
| Leukopenia | 16 (35.6) | 5 (11.1) | 20 (44.4) | | 7 (15.6) |
| Fever | 12 (26.7) | 4 (8.9) | 8 (17.8) | | 3 (6.7) |
| Inappetence | 10 (22.2) | 2 (4.4) | 19 (42.2) | | 8 (17.8) |
| Elevated alanine aminotransferase | 10 (22.2) | 0 | 7 (15.6) | | 0 |
| Elevated Glutamic oxaloacetic transaminase | 10 (22.2) | 0 | 8 (17.8) | | 0 |
| Constipation | 7 (15.6) | 0 | 8 (17.8) | | 2 (4.4) |
| Fatigue | 7 (15.6) | 0 | 7 (15.6) | | 0 |
| Rash | 6 (13.3) | 2 (4.4) | 11 (24.4) | | 1 (2.2) |
| Mouth ulcer | 3 (6.7) | 0 | 3 (6.7) | | 0 |
| Infection | 1 (2.2) | 0 | 3 (6.7) | | 2 (4.4) |
| Diarrhea | 1 (2.2) | 0 | 3 (6.7) | | 0 |

**Table S5.**

**Distributions of Adverse Events in CIK-CT Group**.

| **Adverse Event** | **CIK cells** | | | **Chemotherapy** | |
| --- | --- | --- | --- | --- | --- |
|  | **Any Grade** | **Grade 3-4** | | **Any Grade** | **Grade 3-4** |
|  | **No.(%)** | | | | |
| Anemia | 0 | 0 | 29 (64.4) | | 1 (2.2) |
| Nausea | 0 | 0 | 26 (57.8) | | 3 (6.7) |
| Vomit | 0 | 0 | 23 (51.1) | | 3 (6.7) |
| Thrombocytopenia | 0 | 0 | 18 (40.0) | | 7 (15.6) |
| Leukopenia | 0 | 0 | 16 (35.6) | | 5 (11.1) |
| Fever | 4 (8.9) | 1 (2.2) | 8 (17.8) | | 3 (6.7) |
| Inappetence | 0 | 0 | 10 (22.2) | | 2 (4.4) |
| Elevated alanine aminotransferase | 0 | 0 | 10 (22.2) | | 0 |
| Elevated Glutamic oxaloacetic transaminase | 0 | 0 | 10 (22.2) | | 0 |
| Constipation | 0 | 0 | 7 (15.6) | | 0 |
| Fatigue | 0 | 0 | 7 (15.6) | | 0 |
| Rash | 1 (2.2) | 0 | 5 (11.1) | | 2 (4.4) |
| Mouth ulcer | 0 | 0 | 3 (6.7) | | 0 |
| Infection | 0 | 0 | 1 (2.2) | | 0 |
| Diarrhea | 0 | 0 | 1 (2.2) | | 0 |

**Table S6.**

**List of Investigators and Randomized Patients.**

| **Site name** | **Principal investigator** | **Eligible patients (N = 100)** | **Randomized patients** | |
| --- | --- | --- | --- | --- |
|  |  |  | **CIK-CT group (N = 45)** | **CT group (N = 45)** |
| Tianjin Medical University Cancer Institute and Hospital, Tianjin 300060, China | Xiubao Ren | 33 | 15 | 16 |
|  | Xinwei Zhang |  |  |  |
|  | Liang Liu |  |  |  |
| Affiliated Cancer Hospital of Zhengzhou University & Henan Cancer Hospital, Zhengzhou 450008, Henan, China, | Quanli Gao | 13 | 5 | 6 |
|  | Lingdi Zhao |  |  |  |
|  | Benling Xu |  |  |  |
| Third Affiliated Hospital of Soochow University, Changzhou 213003, Jiangsu, China | Jingting Jiang | 12 | 5 | 5 |
|  | Chen Wu |  |  |  |
|  | Xiao Zheng |  |  |  |
| Shanxi Bethune Hospital, Taiyuan 030032, Shanxi, China | Junping zhang | 11 | 6 | 4 |
|  | Huijing Feng |  |  |  |
|  | Xiaoling Yang |  |  |  |
| Third Affiliated Hospital of Kunming Medical University, Kunming 650118, Yunnan, China | Xin Song | 11 | 5 | 5 |
|  | Hong Yao |  |  |  |
|  | YuanBo Xue |  |  |  |
| First Hospital of Jilin University, Changchun 130021, Jilin, China | Jiuwei Cui | 9 | 4 | 5 |
|  | Xiao Chen |  |  |  |
|  | Lai Qian |  |  |  |
| Fujian Cancer Hospital & Fujian Medical University Cancer Hospital, Fuzhou 350014, Fujian, China | Yunbin Ye | 6 | 3 | 2 |
|  | Yunjian Huang |  |  |  |
|  | Shuping Chen |  |  |  |
| Fourth Hospital of Hebei Medical University, Shijiazhuang 050011, Hebei, China | Zhiyu Wang | 5 | 2 | 2 |
|  | Zheng Wu |  |  |  |
|  | Xing Li |  |  |  |

Abbreviations: CIK-CT, CIK cell immunotherapy plus chemotherapy; CT, chemotherapy.
